# Reproductive and Developmental Biology of *Acroclisoides sinicus*, a Hyperparasitoid of Scelionid Parasitoids

**DOI:** 10.3390/biology10030229

**Published:** 2021-03-16

**Authors:** Lucrezia Giovannini, Giuseppino Sabbatini-Peverieri, Patricia Glynn Tillman, Kim Alan Hoelmer, Pio Federico Roversi

**Affiliations:** 1 CREA—Research Centre for Plant Protection and Certification, I-50125 Florence, Italy; lucrezia.giovannini@crea.gov.it (L.G.); piofederico.roversi@crea.gov.it (P.F.R.); 2 USDA-ARS—Southeast Watershed Research Unit, Tifton, GA 31793, USA; glynn.tillman@usda.gov; 3 USDA-ARS—Beneficial Insects Introduction Research Unit, Newark, DE 19713, USA; kim.hoelmer@usda.gov

**Keywords:** *Halyomorpha halys*, *Trissolcus japonicus*, *Trissolcus mitsukurii*, Scelionidae, Eupelmidae, biological control

## Abstract

**Simple Summary:**

*Acroclisoides sinicus* is a pteromalid of Asian origin that has recently been detected in Europe and North America. It has been frequently found in association with several scelionid and eupelmid primary parasitoids of hemipteran eggs, including the brown marmorated stink bug *Halyomorpha halys*, leading us to suspect that *A. sinicus* is an obligate or facultative hyperparasitoid. Laboratory tests were conducted using pentatomid egg masses (*H. halys*, *Acrosternum heegeri*, and *Dolycoris baccarum*) parasitized by primary parasitoids, including *Trissolcus japonicus*, *Trissolcus mitsukurii*, *Telenomus* sp., *Anastatus bifasciatus*, and unparasitized *H. halys* egg masses, to test this hypothesis. Our studies confirmed that *A. sinicus* is an obligate hyperparasitoid of the pupal stage of scelionid primary parasitoids but not of eupelmid ones, at least under the tested conditions.

**Abstract:**

*Acroclisoides sinicus* (Hymenoptera: Pteromalidae) was described in 1988 from China, but recent findings in Europe and North America within the framework of *Halyomorpha halys* (Hemiptera: Pentatomidae) biological control indicate a Holarctic distribution. The few records and fragmented information on *A. sinicus* are derived from generic observations of other species belonging to the same genus, and its biological and ethological traits are still completely unexplored. It was suspected to be a facultative or obligate hyperparasitoid of many egg parasitoid species (e.g., Scelionidae and Eupelmidae), especially those parasitizing Pentatomidae eggs. Laboratory colonies of *A. sinicus* were established from specimens collected in the field in Europe and the USA, which allowed us to investigate for the first time the life traits of this somewhat enigmatic species. Our studies confirmed the obligate hyperparasitoid hypothesis for species of Scelionidae but not of Eupelmidae. Laboratory studies revealed that *A. sinicus* is extremely selective in its host recognition as only the pupal stage of its host species is exploited for parasitization. Taking into consideration its hyperparasitoid habit, the adventive *A. sinicus* populations in Europe and North America may potentially be severe threats to pentatomid natural control as new components in the trophic chain of pentatomids and their parasitoid guilds.

## 1. Introduction

Many members of the hymenopteran family Pteromalidae, one of the principal taxa of the superfamily Chalcidoidea, are difficult to identify taxonomically, and their biological and ethological traits are often poorly known [1]. The members of this family are distributed worldwide and most species are primary or secondary parasitoids of a great variety of insect species, and therefore play an important role in the natural control of pests [2,3,4]. In particular, species of the genus *Acroclisoides* Girault and Dodd, which are distributed in Afrotropical, Australian, and South Asian regions, are thought to be facultative or obligate hyperparasitoids, principally of hemipteran eggs of the families Pentatomidae and Scutelleridae [5,6]. Primary parasitoids of the family Scelionidae appear to be the most frequent associations, but species of *Acroclisoides* also are associated with species of Eupelmidae [3,4,7]. Despite attempts to study the biology of *Acroclisoides* species, the relationships with their hosts still remains largely unclear [7].

The Asian species *Acroclisoides sinicus* (Huang and Liao) (Hymenoptera: Pteromalidae) was recently discovered in central Europe (Northern Italy and Switzerland) and in North America (USA and Canada) during field surveys of *Halyomorpha halys* (Stål) (Hemiptera: Pentatomidae) [8]. These new findings show not only an Oriental [9,10,11] but also a Holarctic distribution of this species.

In Europe, *A. sinicus* was found mainly associated with *H. halys* and its parasitoid guild, i.e., the native *Anastatus bifasciatus* (Geoffroy) (Hymenoptera: Eupelmidae), and the two adventive exotic species *Trissolcus japonicus* (Ashmead) (Hymenoptera: Scelionidae) and *Trissolcus mitsukurii* (Ashmead) (Hymenoptera: Scelionidae) [8]; additional European records reveal associations also with the pentatomid *Palomena prasina* L. (Hemiptera: Pentatomidae) and the predatory pentatomid *Arma custos* (Fabricius) (Hemiptera: Pentatomidae) [8,12]. In the USA (Georgia, Alabama, and Maryland) and Canada, *A. sinicus* was found associated with at least four different pentatomid species (*H. halys*, *Chinavia hilaris* (Say), *Euschistus* sp., and *Brochymena* sp.) and in combination with three primary egg parasitoids, the eupelmid *Anastatus reduvii* (Howard) and the two scelionids, *Trissolcus edessae* Fouts and *Trissolcus euschisti* (Ashmead) [8]. In addition, *A. sinicus* males and females have emerged from *Nezara viridula* (L.) (Hemiptera: Pentatomidae) egg masses parasitized by *Trissolcus basalis* (Wollaston) (Hymenoptera: Scelionidae) and *H. halys* egg masses parasitized by *Telenomus podisi* Ashmead (Hymenoptera: Scelionidae) collected from the field in the USA (PGT, unpublished data).

Biological and ethological traits of *A. sinicus* are still completely unknown and our work reports the first data on its biology under laboratory conditions. In this study, we hypothesized the possible hyperparasitoid habitus of this pteromalid species in relation to *T. japonicus* and *T. mitsukurii,* two Asian species that are now adventive in Europe, as primary parasitoids of *H. halys* [13,14]. The European native species *A. bifasciatus,* a primary egg parasitoid of pentatomids, was included in our study due to its frequent association with *A. sinicus* in the same egg masses. Additional observations were made of *A. sinicus* parasitizing a native European *Telenomus* sp. as a primary parasitoid of *Acrosternum hegeeri* Fieber (Hemiptera: Pentatomidae); and of *A. sinicus* attacking *T. mitsukurii* as a primary parasitoid of the Palearctic pentatomid *Dolycoris baccarum* L. (Hemiptera: Pentatomidae). Moreover, the features of the immature stages of Pteromalidae are poorly known [15,16]. We are not aware of any papers to date characterizing immature stages of any *Acroclisoides* species; therefore, we schematically describe the juvenile development of *A. sinicus* in this work.

## 2. Material and Methods

### 2.1. Origin of the Insects for Studies in Europe

*Acroclisoides sinicus* were reared from *H. halys* egg masses collected during the summer of 2019 at infested sites in northeastern Italy [8,13]. Field-collected egg masses were reared in glass tubes (15 cm long and 2 cm wide, closed on both ends with a plastic net of 250-μm mesh) and maintained in the climatic chambers at standard conditions of 26 °C, 60% RH, and 16:8 L:D until egg hatch or parasitoid emergence. Identification of adult parasitoids was based on recent taxonomic literature [8,17]. The adults of *A. sinicus* were separated and placed in glass tubes, and reared in the climatic chambers under standard conditions using pure honey droplets as food. Voucher specimens of *A. sinicus* were deposited in CREA laboratory collections and are available upon request.

*Trissolcus mitsukurii* and *A. bifasciatus* were obtained from laboratory colonies at CREA that originated from *H. halys* egg masses from infested field sites, while the *T. japonicus* colony was established with specimens supplied by the USDA-ARS Beneficial Insects Introduction Research Unit, in Newark, Delaware, USA (strain collected in Beijing, China) and imported into Italy under quarantine conditions and authorization number for laboratory studies DG/DISR/DISR05/0013647-19/04/2018. All colonies of the egg parasitoids were maintained on fresh *H. halys* egg masses (age < 24 h). Adult parasitoids were fed with pure honey droplets replenished twice a week. Adults and parasitized host eggs were reared in glass tubes in climatic chambers under the standard conditions mentioned previously.

*Telenomus* sp. (identified as *Telenomus* sp. 1, Francesco Tortorici collection, University of Turin, Italy) were obtained from egg masses of *A. heegeri* collected occasionally in 2020 during entomological field trips. A *Telenomus* sp. colony was reared and maintained under the same conditions as the other egg parasitoids but using its natural host, *A. heegeri*.

The *H. halys* colony originated from individuals collected at infested sites in northern Italy in 2019 and 2020. Specimens were maintained in rearing cages (BugDorm^®^ 4F4545 insect rearing cage, MegaView Science, Taiwan) using fresh fruits (apples, kiwi), vegetables (carrots, green beans), potted plants (soybean), and seeds (peanuts and soybean) as food; water was provided with wet cotton. The colony of *A. heegeri* and *D. baccarum* was established by collecting adults in the field during the 2019 and 2020 growing season, and reared in the lab under the same conditions as *H. halys*. Fresh pentatomid egg masses were collected daily from the colonies for the tests.

### 2.2. Establishment of Acroclisoides sinicus Laboratory Colonies in Europe

To test whether *A. sinicus* was a hyperparasitoid, we established lab-reared colonies of the primary parasitoids *T. japonicus, T. mitsukurii*, and *A. bifasciatus*. Fresh *H. halys* egg masses were offered to females of the three parasitoids for primary parasitization. Considering the limited information available on *A. sinicus* reproductive biology, *H. halys* egg masses (n = 15 per species) were first parasitized by the primary parasitoids and after 24 h offered to females of *A. sinicus* that had emerged from the egg masses collected in the field. To test the combination of *A. sinicus* with the two *Trissolcus* species, primary-parasitized egg masses were exposed to groups of three females of *A. sinicus* for 9 days (since under test conditions *Trissolcus* adults begin emerging from *H. halys* host eggs after 10 days of juvenile development in males and 11 days in females). To obtain an *A. sinicus* colony on *A. bifasciatus*, primary-parasitized egg masses were exposed for 20 days to females of *A. sinicus* (*Anastatus* adults begin emerging from *H. halys* eggs 22 days after oviposition under test conditions). In all combinations, after exposure to *Acroclisoides*, the *H. halys* egg masses were maintained in climatic chambers under standard rearing conditions and checked daily for parasitoid emergence. Any newly emerged adults were removed as soon as possible to prevent intra- and interspecific interference. These F_1_ generations of *A. sinicus* adults were used to produce the next five generations in order to obtain large rearing colonies maintained on the different primary parasitoids.

### 2.3. Rearing Acroclisoides sinicus in the USA under Laboratory and Field Conditions

*Acroclisoides sinicus* was reared on *H. halys* egg masses parasitized by *T. euschisti*. Each of these species originated from individuals collected from infested sites in the southeastern USA in 2019 and 2020 (parasitoids only). The colony of *H. halys* was maintained in rearing cages (27.9 cm long × 26.7 cm wide × 20.3 cm tall) on whole bean pods, apple slices, and raw peanuts at the USDA, ARS Southeastern Watershed Unit in Tifton, GA; knit cloth (97% cotton, 3% spandex) (Jo-Ann Stores, LLC, Hudson, OH, USA) was used as a substrate for oviposition [18]. A *T. euschisti* colony was maintained in individual 25 × 150-mm culture tubes (Kimble^®^, Rockwood, TN) containing honey and raisins. Refrigerated egg masses (≤12 h old when placed in a refrigerator with a temperature range of 2.8–3.3 °C for 24 h) were used for hosts. Eggs were refrigerated to eliminate emergence of first-instar *H. halys*, thus avoiding possible feeding by them on parasitized eggs. Individual female *T. euschisti* were provided with 2–3 *H. halys* egg masses for oviposition. After exposure to females, egg masses were held in a walk-in environmental chamber at 25 °C ± 2.0 °C, 70 ± 10%RH, and 16:8 L:D until adult emergence. The first step in the process towards rearing *A. sinicus* was to offer a *H. halys* egg mass to two *T. euschisti* females for 2 days in a culture tube. In 2019, 2-, 3-, 4-, 6-, and 8-day-old *T. euschisti* parasitized egg masses (n = 10 for 2, 3, and 4 days; n = 25 for 6 and 8 days) were exposed to a single *A. sinicus* female for 72 h in a culture tube. Only one female was placed in a culture tube because females killed additional females. To start a new colony in 2020, individual 5-day-old *T. euschisti-*parasitized egg masses (n = 30) were offered single *A. sinicus* females for 72 h in a culture tube. After exposure to females, egg masses were held in a walk-in environmental chamber at the same settings above until adult emergence. These *A. sinicus* adults were used to produce four generations.

In a preliminary timed exposure test in the field in 2020, lab-reared refrigerated *H. halys* egg masses were used as sentinels to provide hosts for the detection of resident parasitoids in sassafras (*Sassafras albidum* (Nutt.) Nees) for 3, 4, 5, and 6 days. In the laboratory, a portion of the knit cloth with an egg mass was cut from the cloth. Any damaged eggs were gently removed from an egg mass when eggs were counted. Each sentinel egg mass was placed on the underside of a sassafras leaf using a small hair clip. Three egg masses were deployed per treatment on 5 Sept. in Prattville, AL. Retrieved egg masses were held in a walk-in environmental chamber at the same settings above until adult emergence. Voucher specimens of *A. sinicus* were deposited in the Florida State Collection of Arthropods, Gainesville, FL, USA.

### 2.4. Acroclisoides sinicus Reproductive Biology Studies in Europe

**Figure 1:**
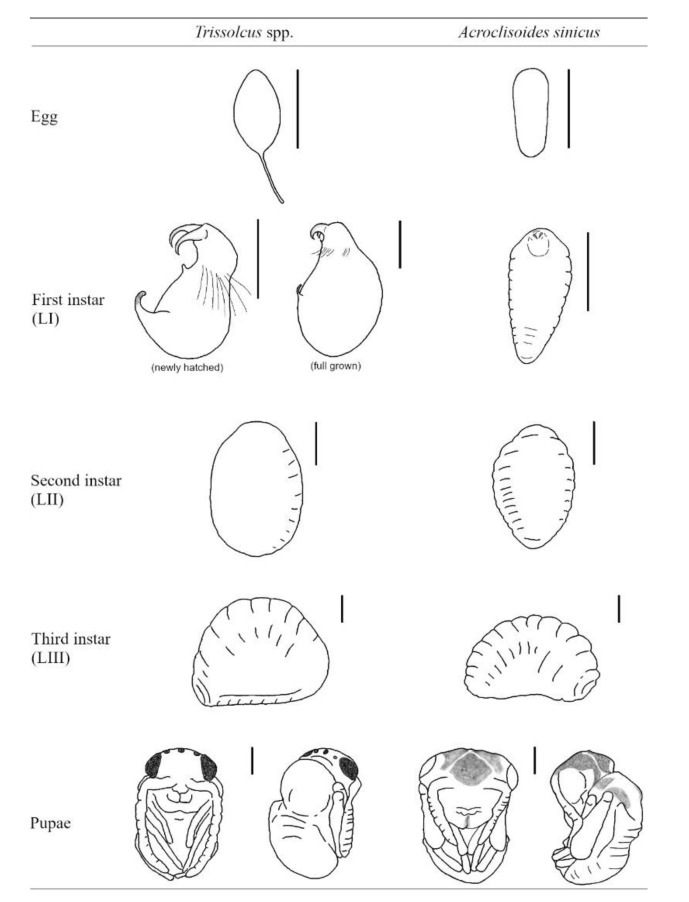
Schematic representation of juvenile developing stages of *Trissolcus* spp. (*T. japonicus* and *T. mitsukurii*) and *Acroclisoides sinicus*. Bars correspond to 200 μm; drawings based on eggs and early larval stages mounted on microslides.

Fresh *H. halys* egg masses were offered for 24 h to females from laboratory colonies of the two primary egg parasitoids, *T. japonicus* and *T. mitsukurii,* to ensure complete parasitization. *Anastatus bifasciatus* was not tested further due to the failure to establish a colony (see the results). After primary parasitization, *H. halys* egg masses were reared in a climatic chamber under standard conditions to obtain a range of development stages of the primary egg parasitoid (see Table 1 for a schematic representation of the experimental designs). Preliminary studies of immature development of both *Trissolcus* species revealed that egg hatch and larval development from the first-instar larva to the mature third-instar larva occurred over 5 days (see the results, Table 2 and Table 3). Morphological differences among these transitional stages are readily recognizable and correspond with the descriptions of four scelionid species, *Telenomus remus* (Nixon), *Telenomus solitus* Johnson, *Typhodites gerriphagus* (Marchal), and *Trissolcus basalis* (Woll.) (see also the results, Figure 1) [19,20,21,22]. The pupal stage occurs approximately from 7 to 9 days, at which time the pharate adult is recognizable (see the results, Table 2 and Table 3). Our preliminary studies revealed that at around day 5 following primary parasitization, the meconium is expelled and is often visible through the cuticle of the host egg (in *T. mitsukurii*, an additional black ring appears below the operculum of the host egg) [8,23]. When this occurs, the immature parasitoid enters, transitionally, the pupal stage.

Tests were conducted in a climatic chamber under standard conditions using single *A. sinicus* females 7–10 days old with no previous ovipositional experience. We assumed females to be mated if males were present in the rearing units prior to collecting females. Females were identified by the presence of a black spot on their fore wing [8]. The black spot is not immediately apparent at female emergence but is clearly visible after 48 h (GL and GSP, personal observations). Additionally, females have a physogastric abdomen enlarged with ovary growth. Egg masses containing primary parasitoids at various developmental stages (1, 3, 5, 7, and 9 days after parasitization) were offered to *A. sinicus* females. *Acroclisoides sinicus* females were exposed to each “age” class for 24 and 72 h, except for the 9-day group, which was exposed only for 24 h, and the 1-day group with an additional treatment of 9 consecutive days of exposure (216 h). Each treatment of parasitoid age/exposure time was replicated 5 times with a single female exposed to an egg mass in each replicate. Three weeks after exposure to *A. sinicus* females, *H. halys* eggs from which neither a nymph or parasitoid emerged, and recorded as pentatomid “dead eggs”, were dissected to detect the cause (dead primary parasitoid, dead *A. sinicus*, dead hemipteran, or undetermined content). To clarify whether *A. sinicus* could develop within an egg mass as a primary parasitoid (rebutting in this case our hypothesis), fresh egg masses of *H. halys* (n = 20) were offered to single females in the same manner as described above and checked daily until eggs hatched or parasitoids emerged.

To determine whether *A. sinicus* exhibits typical haplodiploid sex determination, a common trait in hymenopteran parasitoids, a fresh egg mass (n = 28 eggs) of *H. halys* previously parasitized by *T. mitsukurii* was offered to an unmated female of *A. sinicus* for 9 consecutive days in the same manner as described above. Unmated *A. sinicus* females were obtained from rearing units from which males were removed shortly after emergence and prior to emergence of females, so that females that emerged had no opportunity to copulate.

### 2.5. Acroclisoides sinicus Immature Development Study in Europe

Single egg masses (n = 15) of *H. halys* previously parasitized by *T. mitsukurii* and aged for 5 days to permit immatures of the primary parasitoid to reach the mature larval stage and enter into the pupal stage (see the results) were offered to groups of three *A. sinicus* females (7–10 days old). Three egg masses were exposed for 48 h (“2d”) and three egg masses for 72 h (“3d”) and then dissected. The rest of the egg masses were exposed for 72 h to *A. sinicus* females and then dissected after three (“6d”, 6 days total time), six (“9d”, 9 days total time), and nine (“12d”, 12 days total time) days from the removal of females. Eggs were dissected under a stereomicroscope and the contents carefully inspected. Microslides were prepared when needed for examination of eggs or early larvae. Careful characterization of the three larval instars is critical for assessing the developmental age in species of Pteromalidae. Previous works [15,16] separated larval ages through measurements of morphological features (mandible length and head capsule width) obtained by scanning electron microscopy in conjunction with statistical analysis for *Pteromalus cerealellae* (Ashmead) (Hymenoptera: Pteromalidae) and *Spalangia cameroni* Perkins (Hymenoptera, Pteromalidae), respectively, and recognized three larval instars in both species. In *P. cerealellae* and *S. cameroni*, the second larval instar was approximately 2–3 times bigger in size than the first instar, and the third instar was twice as large as the second one. Being aware of the complexity of instar determination in pteromalids, a detailed description of larval instars of *A. sinicus* will be a forthcoming study, and in our preliminary work, we followed a descriptive and practical approach in referring to three larval instars of *A. sinicus* based on body growth following observations reported in [15,16] (see the results, Figure 1). The immature development study was conducted in a climatic chamber under standard conditions.

### 2.6. Development of Acroclisoides sinicus in Association with Native European Pentatomids

Fresh egg masses of *A. heegeri* (n = 3, 27 eggs in total) were exposed to females of *Telenomus* sp. for 24 h for parasitization. Shortly after primary parasitization, egg masses were exposed for nine days to groups of three female *A. sinicus* (7–10 days old) obtained from the colony reared on *T. mitsukurii*. After exposure, egg masses were held individually in climatic chambers under standard conditions until parasitoids emerged. The same experimental design was used to test the suitability of *D. baccarum* replacing *H. halys* as the primary host in the trophic chain. A fresh egg mass of *D. baccarum* (n = 1, 21 eggs) was exposed to females of *T. mitsukurii* for 24 h for parasitization. After primary parasitization, the egg mass was exposed for nine days to groups of three female *A. sinicus* (7–10 days old) obtained from the colony reared on *T. mitsukurii*. After exposure, the egg mass was held in a climatic chamber under standard conditions until parasitoids emerged.

### 2.7. Acroclisoides sinicus Longevity Tests in Europe

Newly emerged *A. sinicus* adults (males and females) reared on *T. mitsukurii* were isolated individually in glass tubes and provided either with droplets of pure honey or without honey (water was not provided in any case). Specimens were maintained under standard conditions in a climatic chamber without exposure to hosts and checked daily for mortality. Longevity was similarly assessed using *A. sinicus* from the *Telenomus* sp. colony fed with honey.

### 2.8. Polyphenism and Female Egg Load of Acroclisoides sinicus Study in Europe

To determine whether host size influenced the body size of adult *A. sinicus,* we measured the thorax width of *A. sinicus* emerging from *A. heegeri* eggs parasitized by *Telenomus* sp., and from *T. mitsukurii*-parasitized *H. halys* eggs from our laboratory colonies. Morphological characteristics of the pentatomid eggs, including egg height (H), egg width (R = D/2), and width of the operculum (r = d/2), were also measured (n = 12 eggs per host species). Egg volume (V) was estimated using the parabolic barrel formula V = πH(3r^2^ + 4Rr + 8R^2^)/15 [24,25]. The measurements of *A. sinicus* body size and egg volume of pentatomid species were compared with Student’s *t*-test.

To determine ovarial egg load, newly emerged *A. sinicus* females reared on *T. mitsukurii* from the *H. halys* laboratory colony were randomly chosen and placed individually in glass tubes provided with honey droplets and reared in a climatic chamber for 1, 7, 14, or 21 days. After each interval, five *A. sinicus* females were dissected to assess the egg load in their ovaries. Eggs were stained with 1% toluidine blue and counted under a stereomicroscope. *Acroclisoides sinicus* egg dimensions (length and width) were measured on 15 eggs randomly chosen from four females.

## 3. Results

### 3.1. Establishment of Laboratory-Reared Colonies in Europe

**Figure 2:**
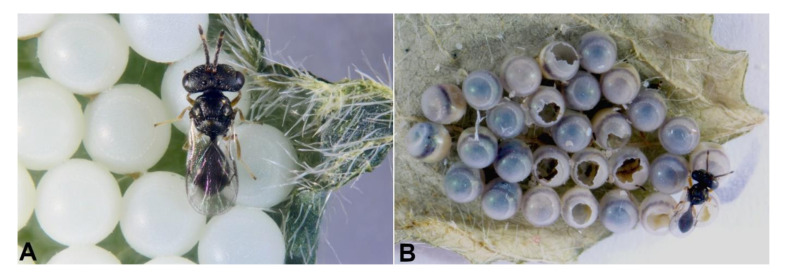
*Acroclisoides sinicus*: (**A**) a female on an egg mass of *Halyomorpha halys*; (**B**) a male is attending the egg mass waiting for newly emerged females to copulate.

Starting from *A. sinicus* females obtained from field-collected *H. halys* egg masses, the establishment of lab-reared colonies was successful on *H. halys* eggs (Figure 2A) parasitized by *T. japonicus* and *T. mitsukurii* but not with those parasitized by *A. bifasciatus.* Despite several attempts to establish a colony on *H. halys* egg masses (n = 15) first parasitized by *A. bifasciatus*, in no case was an *A. sinicus* F_1_ generation obtained under laboratory conditions. From *H. halys* egg masses offered to *A. bifasciatus* for primary parasitization and then to *A. sinicus*, a mean of 56.4 % ± 9.58 *A. bifasciatus* and 37.71 % ± 8.58 *H. halys* nymphs emerged while 5.88 % ± 1.86 of the eggs died. The contents of dissected dead eggs could not be determined and no *A. sinicus* mature larvae, pupae, or pharate adults were observed.

Several *A. sinicus* females that emerged from field-collected *H. halys* egg masses and held in the confined space of glass tubes were frequently observed to display aggressive behaviors, such as biting the antennae, mouthparts, and wings of other females. Aggressivity among females was observed within the rearing tubes but was more frequent when primary parasitized egg masses were available, and host guarding behaviour was observed when potential competitors were present (see Supplementary Materials Video S1). Mating was observed while handling colonies: males emerged (generally one per egg mass) ca. one day before females and lingered on the egg mass waiting for female emergence (Figure 2B); upon female emergence, mating occurred promptly and lasted just a few seconds.

### 3.2. Reproductive Biological Traits of Acroclisoides sinicus in Europe and the USA

*Acroclisoides sinicus* emergence was obtained when egg masses previously parasitized by the two *Trissolcus* species were exposed to females of *A. sinicus* for nine consecutive days after parasitization by the primary scelionid, and when exposing 5- and 7-day-old primary-parasitized egg masses for 72 h to the females (Table 2 and Table 3). No other exposure combinations produced any *A. sinicus* progeny; from those others, only *T. japonicus* or *T. mitsukurii* adults emerged. The single exception occurred for the combination of 7 days × 24 h with *T. mitsukurii* as the primary parasitoid, from which only one male *A. sinicus* emerged (Table 3). In no combination did any *H. halys* eggs hatch. When fresh unparasitized egg masses of *H. halys* were exposed to females of *A. sinicus*, only nymphs of the pentatomid emerged (96.42% ± 0.87 hatched nymphs).

In tests with *T. japonicus* as the primary parasitoid, followed by dissection of dead pentatomid eggs from the 5-, 7-, and 9-day age combinations with the 72-h *A. sinicus* exposure, means (± s.e.) of 7.14 % ± 3.27, 3.77 % ± 1.47, and 8.08 % ± 1.83 per egg mass, respectively, contained pupae and/or adults of *A. sinicus*, while means of 17.85 % ± 4.00, 4.37 % ± 2.07, and 13.82 % ± 0.00 per egg mass, respectively, had undetermined contents. Only in the test with nine days of exposure (1 day × 216 h (9 days)) was it possible to detect dead mature larvae and dead pupae of *T. japonicus* (8.25 % ± 2.28) within dead pentatomid eggs. In all combinations with parasitoid emergence, both sexes of the primary parasitoid *T. japonicus* and *A. sinicus* were represented (Table 2).

In tests with *T. mitsukurii* as the primary parasioid, dissection of the dead pentatomid eggs in the 5- and 7-day age combinations with the 72-h *A. sinicus* exposure revealed that mean values (± s.e.) of 6.23 % ± 1.95 and 1.62 % ± 1.00 per egg mass, respectively, contained pupae and adults of *A. sinicus*; a mean of 2.50 % ± 2.50 per mass had undetermined contents for the 5-day combination. In all combinations with parasitoid emergence, both sexes of the primary parasitoid *T. mitsukurii* and the mated hyperparasitoid were represented (Table 3). Unmated *A. sinicus* females that had been exposed to parasitized *T. mituskurii* immatures produced only male progeny.

In the USA, *A. sinicus* emergence in the laboratory in 2019 was obtained only when exposing 6- and 8-day-old *T. euschisti*-parasitized egg masses for 72 h to the females. For 6-day-old *T. euschisti*-parasitized egg masses, *A. sinicus* males and females emerged from 25% of the egg masses while for 8-day-old parasitized egg masses, *A. sinicus* males and females emerged from 33.3 % of the egg masses. In 2020, both sexes of *A. sinicus* emerged from 100% of the egg masses previously parasitized by *T. euschisti* aged for 5 days and exposed to hyperparasitism for 72 h.

In the preliminary timed exposure test on sassafras in 2020, parasitism of scelionids by *A. sinicus* was significantly influenced by the duration of exposure time of *H. halys* eggs masses used as sentinels on the leaves of this tree. Adult *A. sinicus* did not emerge from egg masses exposed in the field for 2 and 4 days. However, a single *A. sinicus* male emerged from an egg mass exposed for 5 days; 3 *A. sinicus* larvae and 14 larvae and one pupa of *T. podisi* died. Adult *A. sinicus* and *T. podisi* emerged from two egg masses exposed for 6 days. From one egg mass, 6 *A. sinicus* adults (5 females, one male) and 10 *T. podis*i adults (6 females, 4 males) emerged; 2 *A. sinicus* larvae and 4 larvae and one pupa of *T. podisi* died. From the second egg mass, four females of both *A. sinicus* and *T. podisi* emerged; two *A. sinicus* larvae and seven larvae and one pupa of *T. podisi* died; one overwintering third-instar of *A. reduvii* was detected. Because *T. podisi* adults emerged from egg masses deployed in the field for 6 days, it was likely the primary scelionid host species parasitized by *A. sinicus*. The results of the field exposure test agreed with those of the laboratory study.

### 3.3. Acroclisoides sinicus Immature Development Studies in Europe

**Figure 3:**
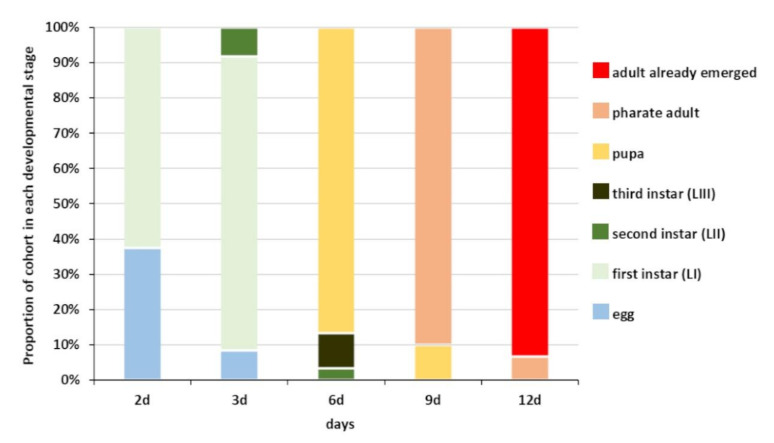
Phenology of *Acroclisoides sinicus* immature development in *Halyomorpha halys* egg masses previously parasitized by *Trissolcus mitsukurii*. Primary-parasitized egg masses were aged for 5 days before exposure to *A. sinicus* females. Hyperparasitized *H. halys* eggs were dissected after the following ageing periods: “2d” = after 48 h of continuous exposure to females; “3d” = after 72 h of continuous exposure to females; “6d” = 72 h exposure + 3 days ageing; “9d” = 72 h exposure + 6 days ageing; “12d” = 72 h exposure + 9 days ageing.

**Figure 4:**
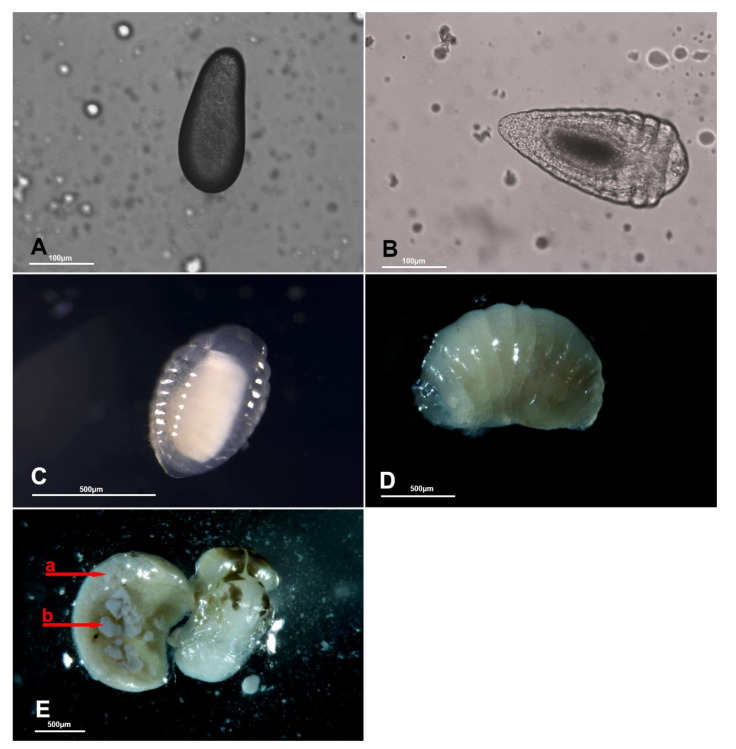
*Acroclisoides sinicus*: (**A**) egg and (**B**) first-instar larva (LI); (**C**) second-instar larva (LI), (**D**) third-instar larva (LIII) and (**E**) pupa; arrow a = large, semicircular meconium of the host species (*Trissolcus* sp.), arrow b = meconium of *A. sinicus* appear as dark-gray grains above the creamy-white meconium of its host.

Using *H. halys* egg masses previously parasitized by *T. mitsukurii* and aged for 5 days (permitting *T. mitsukurii* specimens to reach the transition stage from mature larva to pupa), considerably less than 40% of *H. halys* eggs contained hyperparasitoid eggs after a 48-h initial exposure to *A. sinicus* females (“2d”), and the rest contained immatures of the first larval instar (LI) (Figure 3 and Figure 4A,B). At the end of the 72-h exposure to *A. sinicus* females (“3d”), most of the immatures were in the first larval stage (LI). After a 72-h exposure to *A. sinicus* females and successive rearing of the hyperparasitized *H. halys* egg masses, the following development stages were observed: after three days of rearing (“6d”) most of the immatures were pupae with a few remaining as second and third instars (LII and LIII, respectively) (Figure 3 and Figure 4C–E). After 6 days of rearing (“9d”), most of the specimens were in transition between dark pupae and pharate adults; after 9 days of rearing (“12d”), most of the adults had already emerged. A schematic representation of the immature stages of parasitoids is shown in Figure 1.

**Figure 5:**
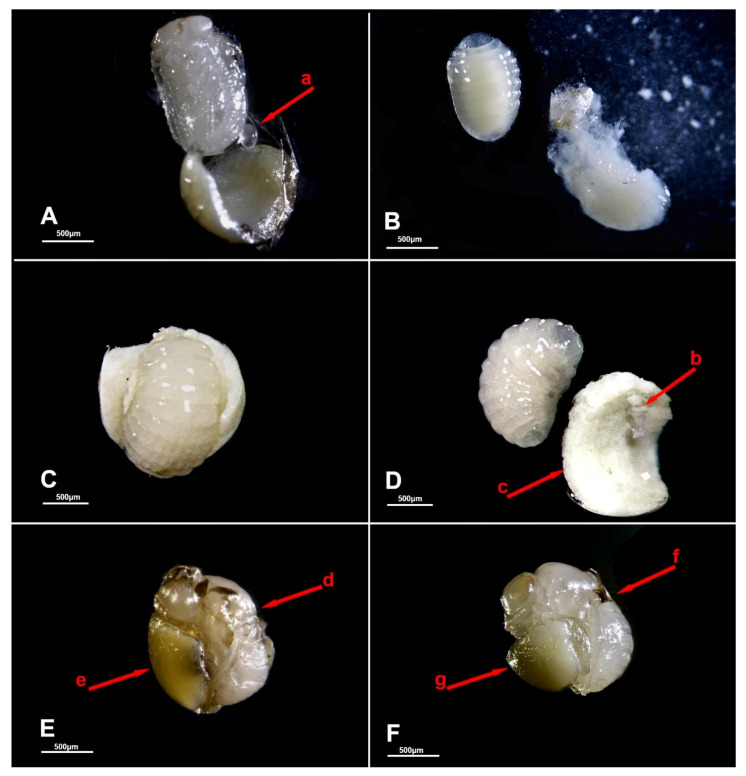
*Acroclisoides sinicus* dissected from *Halyomorpha halys* eggs (eggs dissected and chorion removed): (**A**) first-instar larva (LI, arrow a) adhering externally to its host, the pupa of *Trissolcus japonicus* and its meconium (partially separated for viewing); (**B**) third-instar larva (LIII) and residues of its host after complete feeding; (**C**) third-instar larva (LIII) adhering to meconium of its host *Trissolcus mitsukurii*; (**D**) meconium of the host (arrow c) and first traces of *A. sinicus* meconium (arrow b) (partially separated for viewing); (**E**) pupa of *A. sinicus* (arrow d) adhering to the meconium of its host (arrow e) and displacing its volume within the *H. halys* egg; (**F**) *T. mitsukurii* pupa (arrow f) and its meconium (arrow g) when not parasitized by *A. sinicus*.

Eggs of *A. sinicus* are typically hymenopteriform (spindle-shaped) with a smooth surface, while those of *Trissolcus* species are stalked (with a tube-like extension at one end) [26]. All larval instars of *A. sinicus* are typically hymenopteriform (spindle-shaped, body segmented) with two spur-like mandibles, whereas in scelionids, the first-instar larva is teleaform (body segmentation not visible, cephalothorax separated from the abdomen by a constriction, strong mandibles very large and curved, sclerotized caudal process) and the older instars are hymenopteriform [26] (Figure 1). Dissections of *T. mitsukurii* hyperparasitized by *A. sinicus* in *H. halys* eggs revealed that this hyperparasitoid is an ectoparasitoid, with the first larval stage starting to feed on the body of its host and eventually completely consuming it (Figure 5A,B). Eventually the *A. sinicus* mature larvae (and successively the pupae) completely occupies the space within the *H. halys* egg (Figure 5C–E), which would have been occupied otherwise by the pupae of its *Trissolcus* host (Figure 5F). Traces of meconium of *A. sinicus* expelled by the mature larvae before pupating are clearly visible close to the meconium of its host (Figure 4E and Figure 5D).

### 3.4. Development of Acroclisoides sinicus in Combinations with European Species

**Figure 6:**
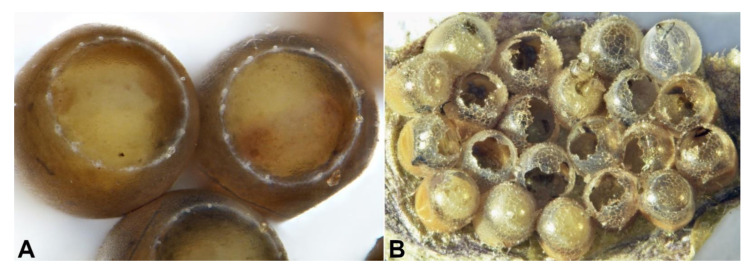
*Acrolisoides sinicus*: (**A**) pupae inside the eggs of *Acrosternum heegeri* previously parasitized by *Telenomus* sp., and (**B**) *A. sinicus* adult emergence holes from the eggs of *Dolycoris baccarum* primary-parasitized by *Trissolcus mitsuklurii*.

From the three egg masses of *A. heegeri* previously parasitized by *Telenomus* sp. and then exposed to *A. sinicus* females, three male and seven female *A. sinicus* emerged from one egg mass (Figure 6); from the other 2 egg masses, 21 specimens of *Telenomus* sp. emerged. From the single egg mass of *D. baccarum* previously parasitized by *T. mitsukurii* and then exposed to females of *A. sinicus,* 2 male and 10 female *A. sinicus* emerged, one pentatomid egg hatched, and 8 eggs were dead with no emergence.

### 3.5. Acroclisoides sinicus Longevity Tests in Europe

Honey-fed females of *A. sinicus* developing on *H. halys* egg masses previously parasitized by *T. mitsukurii* lived for a mean of 45.55 days (±5.09 s.e., n = 11), and males survived for 22.5 days (±3.28 s.e., n = 10). Starved females survived for only 5.4 days (±0.16 s.e., n = 10). Honey-fed females of *A. sinicus* developing on *A. heegeri* egg masses previously parasitized by *Telenomus* sp. lived for 26.0 days (±2.51 s.e., n = 6), and males lived for 16.0 days (±2.0 s.e., n = 2).

### 3.6. Polyphenism and Female Egg Load of Acroclisoides sinicus

**Figure 7:**
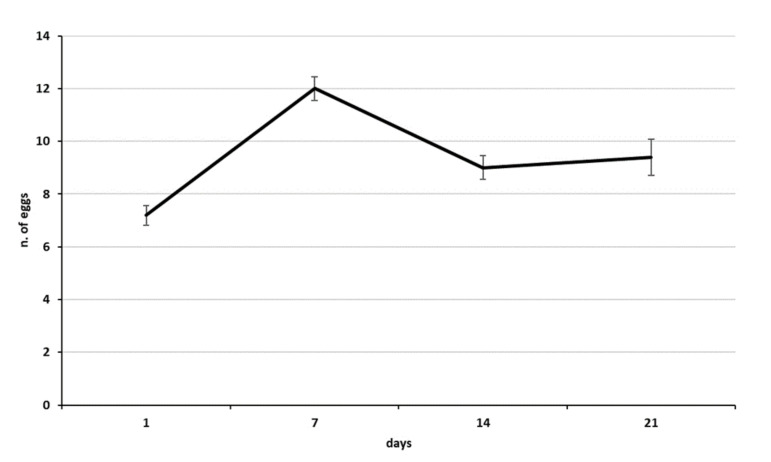
Mean egg load in ovaries of *Acroclisoides sinicus* from the first day after emergence throughout 3 weeks of adult life (females emerged from *Halyomorpha halys* egg masses first parasitized by *Trissolcus mitsukurii*).

Adults of both *A. sinicus* sexes that emerged from *H. halys*+*T. mituskurii* were larger then those that emerged from *A. heegeri*+*Telenomus* sp. The thorax width of *A. sinicus* from both host species (616.22 μm ± 6.19 s.e. from *H. halys*; 451.80 μm ± 6.56 s.e. from *A. heegeri*) was significantly different (*t* = 13.48, *df* = 25, *p* < 0.0001). Eggs of the two pentatomid hosts also varied in volume: *H. halys* eggs were significantly larger (*t* = 27.95, *df* = 20, *p* < 0.0001) than those of *A. heegeri* (1.48 and 0.61 mm^3^, respectively). The egg load in the ovaries of females that emerged from *H. halys* eggs first parasitized by *T. mitsukurii*, without ovipostional experience, varied from 7 to 12 eggs (Figure 7). The mean length and width of *A. sinicus* eggs were 0.262 mm (±0.003 s.e.) and 0.106 mm (±0.002 s.e.), respectively.

## 4. Discussion

The life cycle of *A. sinicus* is poorly known from the literature, but our laboratory study provides important details about its biology that will be valuable for planning more detailed biological studies in the future, such as studies of trophic host–parasitoid chains. The frequent association with different species of field-collected pentatomid egg masses worldwide suggested that this species could be an obligate or facultative hyperparasitoid of primary egg parasitoid species belonging to the genera *Trissolcus*, *Telenomus*, and *Anastatus* [7,8]. Our results confirmed the hypothesis of obligate hyperparasitism by *A. sinicus* of several *Trissolcus* and *Telenomus* species present in Europe and the USA, and it also explains several of the species associations reported in the literature. However, we were not able to rear *A. sinicus* using *A. bifasciatus* as a primary parasitoid, suggesting that this eupelmid species may not be a suitable host, at least under laboratory conditions. Since both these parasitoid species have emerged from field-collected egg masses, their interactions necessitate further studies. The absence of parasitoid progeny after offering females unparasitized egg masses of *H. halys* confirmed that *A. sinicus* is unable to be a primary parasitoid of *H. halys*, at least under our test conditions. Along with several other parasitoid associations recently documented worldwide [8,12], and the results of our laboratory tests using different hosts with primary parasitoids, we conclude that *A. sinicus* is an obligate hyperparasitoid of scelionids but not of eupelmids. However, further tests are needed for a definitive assessment under a wider range of laboratory conditions.

There is a very precise parasitization window for *A. sinicus* females to deposit their own eggs in their hosts to ensure their development, i.e., the time frame represented by the transition from mature larva and meconium expelling to pupal stages, or perhaps only the pupal stage of its host. Production of *A. sinicus* offspring was observed only in primary parasitoid combinations with *T. japonicus* and *T. mitsukurii* pupal stages (*H. halys* egg dissections revealed *A. sinicus* eggs and early instar larvae only associated with the presence of scelionid pupae). This was also demonstrated by the co-presence of the meconium of the primary parasitoid and the hyperparasitoid within the *H. halys* egg; thus, the pentatomid egg shell provides a protected environment for developing *A. sincus* immatures, which feed on sceliond pupae. In all cases so far observed, the meconium of *A. sinicus* is deposited directly on the meconium of its host and not randomly within the pentatomid egg [23]. The olfactory cues in the meconium could assist female *A. sinicus* in their search for hosts and subsequent acceptance, as is known to be the case with many parasitoid and predator species [27]. Progeny production was not observed when larval instars or pharate adults of *Trissolcus* species were offered for parasitization.

Hyperparasitism can occur with different levels of specialization depending on the associations between different host parasitoid and hyperparasitoid species. For example, the eupelmid *A. bifasciatus* is known to be a broadly generalist primary parasitoid, able to exploit both pentatomid and lepidopteran eggs, but it is also a facultative hyperparasitoid of several primary parasitoids at different development stages, including *T. japonicus* [28,29,30]. Many pteromalids are pupal parasitoids as is *A. sinicus*.

It was not possible to clearly define the developmental time for the *A. sinicus* immature stages because progeny production was obtained only by exposing the host for 72 h to parental females. Progeny production was not successful with only a 24-h exposure time except in a single case. The parasitization process likely occurs between the second and third day of host exposure, thus the developmental time of *A. sinicus* should be between 10 and 11 days. The single exception of progeny production after a 24-h host exposure resulted in a 10-day developmental time and produced a male. A similar developmental time was reported for the pteromalid *P. cerealellae* [15]. Under the same rearing conditions, immature development of *A. sinicus* is similar to that of *Trissolcus* species attacking *H. halys* [31,32].

Males always emerge a day before the females and mating occurs immediately on the egg mass, evidence that sib-mating occurs frequently, as observed for *P. cerealellae* [15]. Sib-mating is also a common behavoir in egg parasitoids of pentatomids, such as *T. japonicus* and *Gryon pennsylvanicum* (Ashmead) (Hymenoptera: Scelionidae) [32,33].

As shown by our preliminary studies on immature development of *T. japonicus* and *T. mitsukurii*, the first larval stages are teleaform, which is typical for scelionids, and mandibles are abnormally developed compared to the last larval instar. Different types of mandibles may reflect different behaviors by first-instar larvae of these parasitoids. *Trissolcus japonicus* and *T. mitsukurii* may be adapted to develop in a more competitive environment, fighting for resources with competitors in the *H. halys* parasitoid guild. In contrast, *A. sinicus* larvae apparently develop in a less competitive environment, attacking a host that is generally less active, i.e., the pupa of its scelionid host. In all cases, the purpose of mandibles of such larvae may be to puncture the host integument and imbibe fluid food oozing from the host body, as described for *S. cameroni* [16].

When provided with carbohydrate in the form of honey, *A. sinicus* adults live a long time in the lab, similar to *T. japonicus* or *A. bifasciatus* [34,35]. Without food *A. sinicus* can survive for several days, much longer than *Trissolcus* [34]. Longevity seems to be host dependant, for *A. sinicus* females developing on the *H. halys*–*T. mitsukurii* combination lived twice as long as those developing on the *A. heegeri*–*Telenomus* combination. These differences could due to the quantity and/or quality of food resources of the different hosts: *A. sinicus* that developed on the *A. heegeri–Telenomus* sp. association are smaller than those that developed on *H. halys–T. mitsukurii* one, and *A. heegeri* eggs are smaller than those of *H. halys*. This was also seen in *T. mitsukurii*, whose adults live longer when they develop on *H. halys* eggs than on *N. viridula* eggs [36,37].

The Asian *A. sinicus* attacks not only several exotic *Trissolcus* species that have become invasive in Europe, but also several European native egg parasitoids, developing in different host associations, such as *Telenomus* sp. developing on *A. heegeri* or *T. mitsukurii* developing on *D. baccarum*. The emergence of *A. sinicus* males and females from field-collected egg masses strongly suggests that that this hyperparasitoid might also attack at least some of the native *Trissolcus* and *Telenomus* species parasitizing a range of pentatomid host eggs in the USA [8], and the development of *A. sinicus* in the laboratory confirms its hyperparasitism behavior of the North American scelionid *T. euschisti*. In addition to the results reported in this paper, rearing records from other field surveys reported the development of *A. sinicus* on (unidentified) parasitoids within the pentatomid genera *Brochymena* sp.*, C. hilaris, Euschistus* sp., *P. prasina*, and *H. halys* ([8] and KAH unpublished data).

Hyperparasitism by *A. sinicus* of scelionids, including *T. japonicus* as shown in our study, could have important consequences for classical biological control of *H. halys* by the release of exotic parasitoids (independently of *T. japonicus* or *T. mitsukurii*). A related question concerns the ecological impact of such hyperparasitoid on the global natural control provided by native egg parasitoid guilds and their role in the ecosystem. The recent discoveries of adventive *A. sincus* in newly invaded areas of Europe and North America suggest that it is likely to become a permanent part of the local fauna, but the impact of this species to the ecosystem of the North American and European fauna is a matter to be assessed in the future [38]. Biological traits presented in our work can explain many aspects of biological and ethological features under field conditions and can be helpful for future planning of research with such complicated parasitoid species, where highly specific optimal timing of host suitability within the entire trophic chain is fundamental for the success of the parasitoid’s reproduction.

## 5. Conclusions

We conclude that *A. sinicus* is an obligate hyperparasitoid of the scelionids *T. japonicus*, *T. mitsukurii*, and *Telenomus* sp. but not of the eupelmid *A. bifasciatus*. Adult emergence of the hyperparasitoid was obtained upon exposing 5- and 7-day-old primary-parasitized egg masses for 72 h to *A. sinicus* females, suggesting that this pteromalid is a highly specific hyperparasitoid exploiting hosts at the pupal stage. The recent findings in Europe and North America of this Asian hyperparasitoid emerging from pentatomids are concerning in that this pteromalid may interfere in the trophic chains that regulate the population dynamics of stink bugs.

## Figures and Tables

**Table 1:** Schematic representation of the experimental studies of *Acroclisoides sinicus* reproductive biology.

Trophic Chain	Pentatomid Eggs		Primary Parasitization		Aged Primary Parasitized Host		Hyperparasitization		Hyperparasitized Egg Masses
	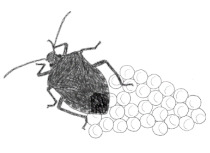	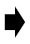	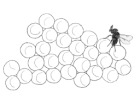	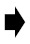	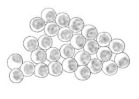	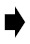	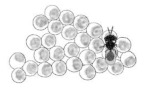	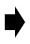	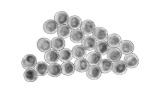
	**Fresh (<24 h) *H. halys* Egg Masses**		***Trissolcus*** **spp. Parasitize Host Eggs**		***Trissolcus*** **spp. Juveniles Develop Inside Its Host**		***A. sinicus*** **Parasitize Its Host *Trissolcus* spp. inside Eggs of *H. halys***		***A. sinicus*** **Juveniles Develop by Feeding on Its Host *Trissolcus* spp. inside *H. halys* Eggs**
**Parasitization tests**	*H. halys* egg masses		Exposure to parasitization for 24 h		Ageing for 1, 3, 5, 7, and 9 days		Exposure for 24 (all treatments) and 72 h (except 9 days ageing) and 216 h (only for 1 day)		Rearing egg masses to emergence of adult parasitoids
**Juvenile development study**	*H. halys* egg masses		Exposure to parasitization for 24 h		Ageing for 5 days		Exposure for 48 h and 72 h		Egg dissection at the end of exposure (48 h = “2d”; 72 h = “3d”)
							Exposure for 72 h		Eggs rearing for additional 3 (“6d”), 6 (“9d”), and 9 days (“12d”) before dissection

*Halyomorpha halys* egg masses were first parasitized by *Trissolcus* spp. (*T. japonicus/T. mitsukurii*) and subsequently exposed to females of *A. sinicus* in different combinations of ageing and exposure intervals. As a control, unparasitized fresh (<24 h) egg masses of *H. halys* were offered to females up to egg hatching (not shown here).

**Table 2:** *Acroclisoides sinicus* reproductive success on egg masses of *Halyomorpha halys* parasitized by *Trissolcus japonicus* (means ± s.e.); LI = first-instar larvae, LII = second-instar larvae, LIII = third-instar larvae.

Exposure Combination (Egg Mass Age/Exposure Time)	*T. japonicus* Development Stage	n. of Eggs/Egg Mass	Adult Emergence Rate (%)		Dead *H. halys* Eggs	Sex Ratio (% Female)	
			*T. japonicus*	*A. sinicus*		*T. japonicus*	*A. sinicus*
1 day × 24 h	Egg	27.00 ± 0.70	93.68 ± 4.22	0.00	6.46 ± 4.23	89.84 ± 2.74	-
1 day × 72 h	Egg–LI	27.50 ± 0.28	99.07 ± 0.92	0.00	1.85 ± 0.00	92.65 ± 2.65	-
3 days × 24 h	LI	27.50 ± 0.50	97.11 ± 2.88	0.00	2.88 ± 0.00	92.27 ± 1.96	-
3 days × 72 h	LI–LII–LIII	25.25 ± 0.85	91.65 ± 4.82	0.00	8.34 ± 4.82	95.99 ± 1.53	-
5 days × 24 h	LIII	27.75 ± 0.25	94.60 ± 3.08	0.00	5.39 ± 3.08	94.32 ± 1.81	-
5 days × 72 h	LIII–pupa	28.00 ± 0.00	59.82 ± 7.95	19.64 ± 5.15	20.24 ± 1.79	88.10 ± 4.76	80.44 ± 8.33
7 days × 24 h	Pupa	26.75 ± 0.75	98.07 ± 1.92	0.00	1.92 ± 0.00	91.38 ± 2.56	-
7 days × 72 h	Pupa–pharate adult	27.40 ± 0.60	89.74 ± 2.88	1.43 ± 1.43	8.11 ± 1.87	89.29 ± 1.24	50.00 ± 0.00
9 days × 24 h	Pharate adult	27.50 ± 0.57	92.75 ± 3.73	0.00	7.24 ± 3.73	95.19 ± 0.79	-
1 day × 216 h	From egg to pharate adult	25.56 ± 1.50	46.48 ± 4.95	23.21 ± 11.08	30.30 ± 6.46	53.50 ± 8.53	66.43 ± 17.34

**Table 3:** *Acroclisoides sinicus* reproductive success on egg masses of *Halyomorpha halys* parasitized by *Trissolcus mitsukurii* (means ± s.e.); LI = first-instar larvae, LII = second-instar larvae, LIII = third-instar larvae.

Exposure Combination (Egg Mass Age/Exposure Time)	*T. mitsukurii* Development Stage	n. of Eggs/Egg Mass	Adult Emergence Rate (%)		Dead *H. halys* Eggs	Sex Ratio (% Female)	
			*T. mitsukurii*	*A. sinicus*		*T. mitsukurii*	*A. sinicus*
1 day × 24 h	Egg	27.50 ± 0.51	100.00	0.00	0.00	61.57 ± 13.43	-
1 day × 72 h	Egg–LI	27.00 ± 0.00	100.00	0.00	0.00	66.07 ± 19.64	-
3 days × 24 h	LI	27.50 ± 0.52	100.00	0.00	0.00	52.98 ± 13.69	-
3 days × 72 h	LI–LII–LIII	26.80 ± 0.73	100.00	0.00	0.00	87.42 ± 5.07	-
5 days × 24 h	LIII	27.50 ± 0.57	95.01 ± 1.35	0.00	4.98 ± 2.88	79.62 ± 4.76	-
5 days × 72 h	LIII–pupa	25.80 ± 0.66	16.90 ± 0.00	33.01 ± 9.14	50.06 ± 10.56	100.00	83.79 ± 7.95
7 days × 24 h	Pupa	28.00 ± 0.57	77.90 ± 18.58	1.23 ± 0.00	22.09 ± 18.58	77.90 ± 18.58	-
7 days × 72 h	Pupa–pharate adult	26.20 ± 1.11	47.21 ± 18.32	27.00 ± 10.51	26.25 ± 17.51	96.87 ± 1.99	61.79 ± 17.25
9 days × 24 h	Pharate adult	27.50 ± 0.57	100.00	0.00	0.00	93.69 ± 1.13	-
1 day × 216 h	From egg to pharate adult	25.40 ± 1.20	14.40 ± 0.00	85.60 ± 14.40	0.00	94.44 ± 0.00	90.69 ± 1.76

## Data Availability

Data archived with Zenodo is available under Creative Commons Attribution 4.0 International (CC BY-NC-ND 4.0).

## Supplementary Materials

Video S1: *Acroclisoides sinicus* life traits: http://doi.org/10.5281/zenodo.4560820.
